# A single cell RNAseq benchmark experiment embedding “controlled” cancer heterogeneity

**DOI:** 10.1038/s41597-024-03002-y

**Published:** 2024-02-02

**Authors:** Maddalena Arigoni, Maria Luisa Ratto, Federica Riccardo, Elisa Balmas, Lorenzo Calogero, Francesca Cordero, Marco Beccuti, Raffaele A. Calogero, Luca Alessandri

**Affiliations:** 1 Department of Molecular Biotechnology and Health Sciences, University of Torino, Torino, Italy; 2 Department of Electronics and Telecommunications (DET), Politecnico di Torino, Torino, Italy; 3 Department of Computer Science, University of Torino, Torino, Italy

**Keywords:** Non-small-cell lung cancer, Gene regulatory networks

## Abstract

Single-cell RNA sequencing (scRNA-seq) has emerged as a vital tool in tumour research, enabling the exploration of molecular complexities at the individual cell level. It offers new technical possibilities for advancing tumour research with the potential to yield significant breakthroughs. However, deciphering meaningful insights from scRNA-seq data poses challenges, particularly in cell annotation and tumour subpopulation identification. Efficient algorithms are therefore needed to unravel the intricate biological processes of cancer. To address these challenges, benchmarking datasets are essential to validate bioinformatics methodologies for analysing single-cell omics in oncology. Here, we present a 10XGenomics scRNA-seq experiment, providing a controlled heterogeneous environment using lung cancer cell lines characterised by the expression of seven different driver genes (EGFR, ALK, MET, ERBB2, KRAS, BRAF, ROS1), leading to partially overlapping functional pathways. Our dataset provides a comprehensive framework for the development and validation of methodologies for analysing cancer heterogeneity by means of scRNA-seq.

## Background & Summary

Genetic and transcriptomic heterogeneity within tumours is crucial in how patients react to treatment. The process of natural selection can result in the development of subpopulation of cells within the tumour that are resistant to drugs. Consequently, the identification and the molecular profiling of such subgroups can provide valuable insights to decipher the tumour evolution. Moreover, the clear identification of tumour cell types can potentially uncover new opportunities for therapeutic intervention. Organoids serve as a potent tool for exploring tumour diversity and drug reactions. These microscopic, self-arranging, three-dimensional structures mimic numerous structural and functional characteristics of their corresponding organs in the body. This adaptable technology has facilitated the creation of innovative human cancer models, enabling the generation of organoids from tumour tissues of individuals with various carcinomas^1^. Organoid technology and breakthroughs in single-cell omics can potentially transform cancer research, providing the capability to comprehensively classify cell types and identify tumour subclones^2^.

Recent applications of single-cell RNA sequencing (scRNA-seq) have yielded new insights into the advancement of cancer, along with a better understanding of how the tumour response to treatment^3–5^. However, pinpointing intratumor genetic heterogeneity and detecting subclones using scRNA-seq is challenging due to the inherent noise in single nucleotide variants (SNVs) derived from scRNA-seq data. Despite this obstacle, considering that the gene activity within tumours is impacted by genetic differences among tumour cells, the classification of cells into subclones and the comprehensive investigation of genetic modifications within each subclone remain essential components of any scRNA-seq investigation in oncology. The analysis of scRNA-seq data to depict and characterise tumour subpopulations massively depends on computation frameworks^6^. However, the overall performance of these tools can be hardly addressed, because of the lack of specifically designed benchmark experiments. For instance, the computational tools addressing genomic aberrations^7,8^ and SNVs^9–11^, had no specifically defined benchmark datasets designed for their assessment and the tools validation were performed either on datasets derived from previous studies^8,10,11^ or using synthetic data^7,9^.

The primary goal of benchmarking studies is to meticulously assess and compare the effectiveness of various methods using thoroughly characterised benchmark datasets. This assessment allows to identify the respective merits of each method and offers guidance on the most suitable choice for a given analysis. Nonetheless, the design and the execution of benchmarking studies require meticulous attention to ensure that the results are both precise and impartial, providing valuable and unbiased insights^12^. As part of the guidelines for the implementation of benchmark experiments, there is the need to select or design representative datasets^12^. Here, we present a multi-purpose benchmark dataset, based on 10XGenomics technology, and designed to address the following challenges:

1. Depicting different subpopulation controlled by different cancer driver genes. This can be achieved using seven unique cell lines, each marked by a specific driver mutation, which are characterised by the presence of partial overlaps in their functional pathways, Fig. 1:
  - PC9, EGFR Δ19, activating mutation;^13^
  - A549, KRAS p.G12S, affecting growth and proliferation;^14^
  - NCI-H1395 (CRL5868), BRAF p.G469A, gain of function mutation providing resistant to all tested MEK + /− BRAF inhibitors;^15^
  - DV90, ERBB2 p.V842I, increasing kinase activity;^16^
  - NCI-H596 (HTB178), MET Δ14, enhancing protection from apoptosis and favouring cellular migration;^17,18^
  - HCC78, encompassing SLC34A2-ROS1 Fusion, controlling signalling pathways, being critical for growth and survival^19^.
  - CCL-185-IG, an A549 isogenic cell line created to model cancer patients with the echinoderm microtubule-associated protein-like 4 (EML4)-anaplastic lymphoma kinase (ALK) fusion oncogene (EML4 exon13; ALK exon20) and sensitive to inhibitors of ALK^20^.

**Fig. 1:**
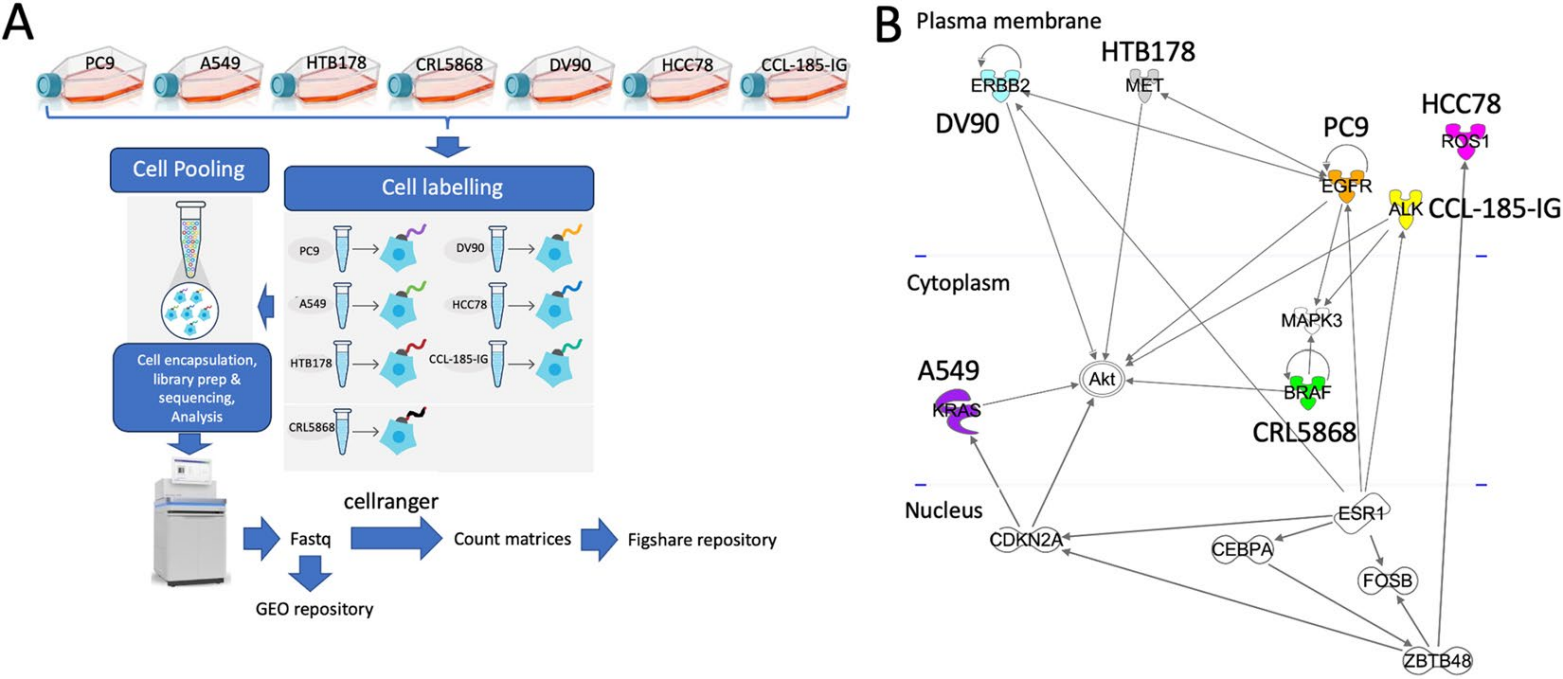
Single cell RNAseq benchmark experiment embedding “controlled” cancer heterogeneity (**A**) Outline the experimental workflow (**B**) Functional relationships among EGFR, ALK, MET, ERBB2, KRAS, BRAF, ROS1 cancer driver genes. The full list of relations is available as figshare repository^26^.
  By employing varying proportions of cells from different cell lines^20^, it will be feasible to mimic the heterogeneity found in real-life scenarios. This approach will enable the assessment of computational tools in their capacity to identify subpopulations effectively.
2. Depicting different subpopulations characterised by having acquired a new driver mutation. A549 (KRAS p.G12S) and CCL-185-IG, could be valuable for evaluating computational tools capabilities to capture subtle variations within cell subpopulations, e.g., those emerging within cancer organoids following drug treatment.
3. Utilising scRNA-seq data from PC9, A549, CCL-185-IG, and NCI-H1395 (CRL5868) cells could serve as a suitable approach to illustrate the connections between EGFR-mutated transcriptomes and the development of osimertinib-resistant non-small cell lung cancer (NSCLC) with secondary molecular driver alterations. These alterations might include ALK fusions or BRAF and KRAS mutations^21^. This dataset could serve as the foundation for assessing the feasibility of predicting the occurrence of distinct secondary molecular driver alterations.
4. The above mentioned seven cell lines provide an ideal environment to develop a new class of computation tools able to depict new hidden driver genes^22^.

The purpose of this dataset is to function as a validation tool for computational methods specialized in the characterization of cancer heterogeneity through single-cell analysis. The fundamental idea behind this dataset entails the utilisation of homogeneous cell lines to generate virtual replicates, ensuring a comprehensive understanding of cell composition heterogeneity.

## Methods

### Cells

NCI-H596 (ATCC-HTB-178), NCI-H1395 (ATCC-CRL-5868), A549 (ATCC-CCL-185) and EML4-ALK Fusion-A549 (ATCC-CCL-185-IG) human lung cancer cell lines were purchased from the American Type Culture Collection. PC9 (CSC-C4619J) human lung cancer cell line was purchased from Creative Bioarray; DV90 (ACC 307) and HCC78 (ACC 563) human lung cancer cell lines were provided from DSMZ Leibniz Institute.

A549 and EML4-ALK Fusion-A549 were maintained in F12Kmedium (ATCC-30-2004), plus heat-inactivated 10% FBS (ATCC-30-2020) and antibiotics-antimycotics (Gibco, # 15240062) and cultured in 5% CO2 at 37 °C.

NCI-H596, NCI-H1395 and PC9 were maintained in RPMI 1640 medium (ATCC-30-2001) plus heat-inactivated 10% FBS (ATCC-30-2020) plus antibiotics-antimycotics (Gibco, # 15240062) and cultured in 5% CO2 at 37 °C.

DV90 were maintained in RPMI 1640 medium (ATCC-30-2001), plus heat-inactivated 10% FBS (ATCC-30-2020), plus 2 mM L-glutamine (ATCC-30-2214), plus 1x non-essential amino acids (M7145, Merck), plus antibiotics-antimycotics (Gibco, # 15240062) and cultured in 5% CO2 at 37 °C.

HCC78 were maintained in RPMI 1640 medium (ATCC-30-2001) plus heat-inactivated 20% FBS (ATCC-30-2020) plus antibiotics-antimycotics (Gibco, # 15240062) and cultured in 5% CO2 at 37 °C.

All cell lines were routinely tested for Mycoplasma using the Mycoalert Mycoplasma detection kit (Lonza). Cells were propagated from the vial supplied by the vendor, divided into aliquots, and preserved under liquid nitrogen. Subsequently, for each cell line, a vial was thawed and expanded through two passages to attain the necessary cell quantity for a 10XGenomics scRNA-experiment.

### 10XGenomics library preparation

To obtain single-cell RNA-seq data, 10x Genomics Chromium Next GEM Single Cell 3’ Kit v3.1 (10X Genomics, Next GEM Single Cell 3’ Kit v3.1 CG00390 Rev C) was used according to the manufacturer’s instructions. Briefly, cultured cells were diluted in PBS, washed, and then incubated with Cell multiplexing oligos (10X Genomics, Cell-plex CG000391 Rev A n 2) according to the manufacturer’s instructions. After washing and prior 10X chip loading, cells were counted and showed high viability (at least 80%) and low level of aggregates. Subsequently, cell-plexed cells were mixed, counted again and then loaded on a Chromium Next GEM chip G (Chromium system, 10X Genomics). Post GEM-RT clean up, cDNA amplification and library construction were performed following manufacturer’s instructions (10X Genomics, Next GEM Single Cell 3’ Kit v3.1 CG00390 Rev C). Libraries quality was determined through TapeStation D5000 ScreenTape (Agilent Technologies). Libraries were quantified both by Qubit 2.0 (ThermoFisher) and QuantStudio 5 System (Applied Biosystems). The library pool was loaded and sequenced on an Illumina® NovaSeq X plus 10B flow-cell (Illumina) at a final loading concentration of 150 pM with a read length configuration of 150PE.

### Counts table generation

Counts table generation and demultiplexing are intertwined in the 10xGenomics 3’ CellPlex protocol. The 10xGenomics 3’ CellPlex protocol presents a versatile solution for sample multiplexing, utilising barcode oligonucleotides linked to a lipid molecule. This protocol allows the combination of up to 12 samples, with sample demultiplexing seamlessly integrated into the counts table generation process, managed by the cellranger program (version 7.0 or higher). For this data set count matrices were generated using 10XGenomics cellranger program (v.7.1.0), with intronic reads included in the counts quantification. Cellranger is available as a Docker container at docker.io/repbioinfo/cellranger.2023.7.1.0. The docker can be accessed with the command:

docker run -v /somewhere_in_your_server/fastq_folders/:/data -v /somewhere_in_your_server/10Xgenomics_reference_folder/:/genomes -it repbioinfo/cellranger.2023.7.1.0 /bin/bash

The analysis can be run using the command:

cellranger multi --id = BE1run1 --csv = /data/multi_gex.csv

The sample sheet multi_gex.csv, required by cellranger is part of the supplementary files available as GEO repository in the GSE243665 series.

The genome reference used from cellranger is refdata-gex-GRCh38-2020-A, which can be retrieved from 10XGenomics repository with the following command:

wget “https://cf.10xgenomics.com/supp/cell-exp/refdata-gex-GRCh38-2020-A.tar.gz”.

## Data Records

The fastq data are available at series in GEO NCBI repository^23^ and at SRA NCBI repository^24^.

## Additional data

As additional data, the count tables, in 10XGenomics sparse matrix format, are also available at figshare repository^25^.

Further supporting information is also available at figshare repository^26^. This figshare repository provides information retrieved from CCLE database (ccle.xlsx) for PC9, A549, NCI-H596 (HTB178), NCI-H1395 (CRL5868), DV90, HCC78. Since CCL-185-IG is an A549 isogenic cell line, it is expected to share with A549 fusion genes and somatic mutations. It also includes driver gene (EGFR, MET, KRAS, BRAF, ERBB2, ALK, ROS1) – downstream direct targets relationships retrieved from IPA (Qiagen, retrieved on June 2^nd^ 2023) and TRRUST^27^ database (drivers_genes_relations.xlsx). The code used to extract the information from CCLE files is also part of this supporting information.

## Technical Validation

The sequencing was done on two lanes of NovaSeq X plus 10B flow-cell. The total sequencing was 2.46 billion reads with a minimum of 71.26% of bases ≥ Q30.

Cellranger analysis did not provide any alert for any of the sequenced cell lines.Table 1 reports the statistics provided for each cell line by cellranger during the generation of the count matrices.

**Table 1:** Cellranger statistics.

Cell line	Cells	Median reads per cell	Median genes per cell	Total genes detected	Median UMI counts per cell
A549	6,898	21,728	2,315	27,584	5,961
CCL-185-IG	6,354	21,493	2,294	26,855	5,935
CRL5868	2,673	17,240	1,607	25,705	4,532
DV90	2,998	21,241	2,160	25,786	5,628
HCC78	2,748	28,423	2,665	25,425	7,808
HTB178	2,965	19,652	2,113	25,281	5,300
PC9	4,492	27,026	2,746	26,881	7,437

**Fig. 2:**
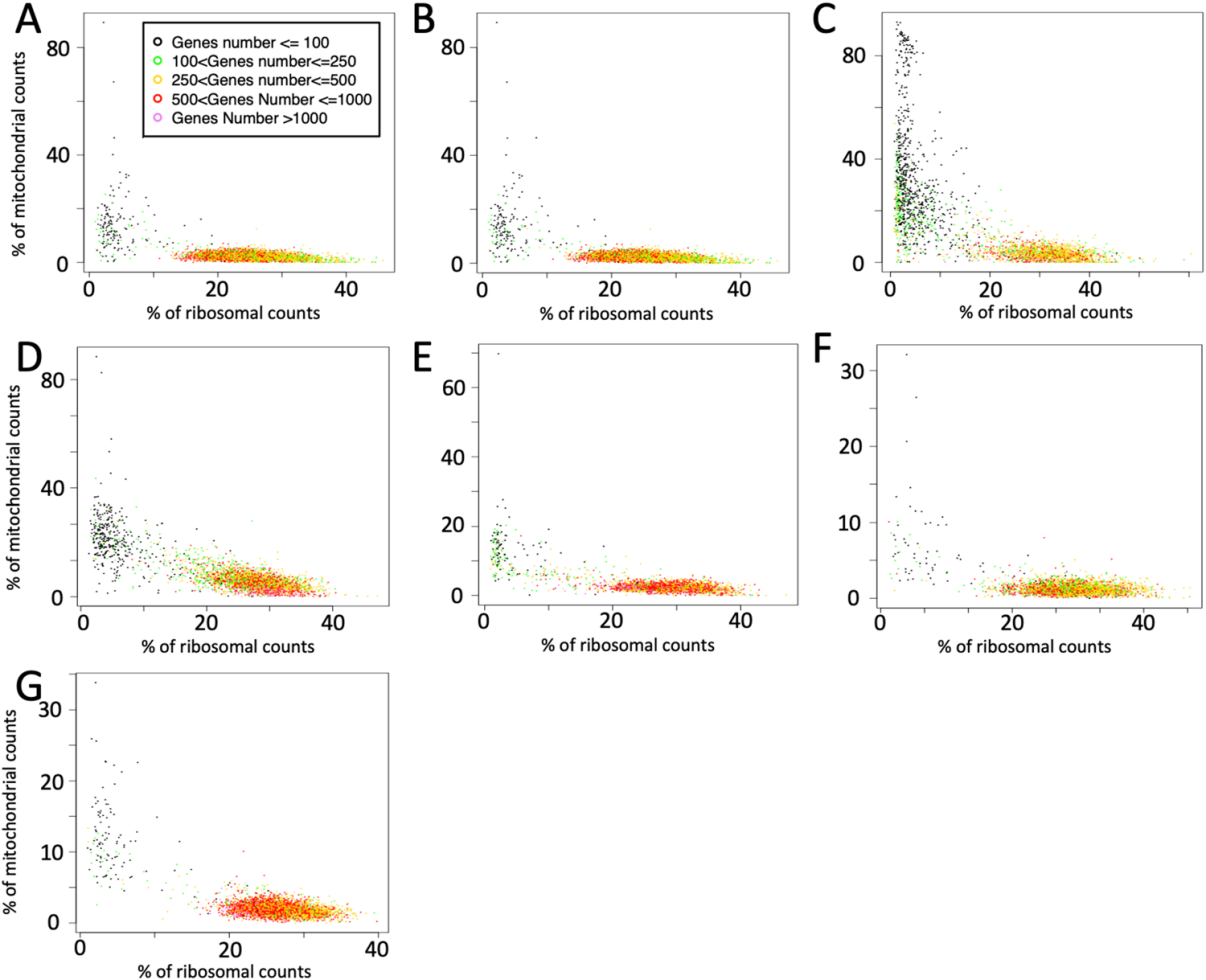
Quality control of the cells. Low quality cells are characterised by having a low number of genes depicted as present, associated with low ribosomal content and high mitochondrial content. (**A**) A549, (**B**) CCL-185-IG, (**C**) CRL5868, (**D**) DV90, (**E**) HCC78, (**F**) HTB178, (**G**) PC9.

We also run a basic QC data analysis using rCASC^28,29^. Specifically, we run mitoRiboUmi plot^30^ (parameters: gtf.name = “Homo_sapiens.GRCh38.99.gtf”, bio.type = “protein_coding”, umiXgene = 3) to depict low quality cells, Fig. 2. Only CRL5868 seem to have a relatively high number of stressed cells^31^. Nevertheless, excluding low quality cells, the total cell decreases from 2673 to 1939. This revised number remains reasonable for the creation of “virtual” organoids, simulating a blend of various cancers subpopulations distinguished by distinct driver genes.

**Fig. 3:**
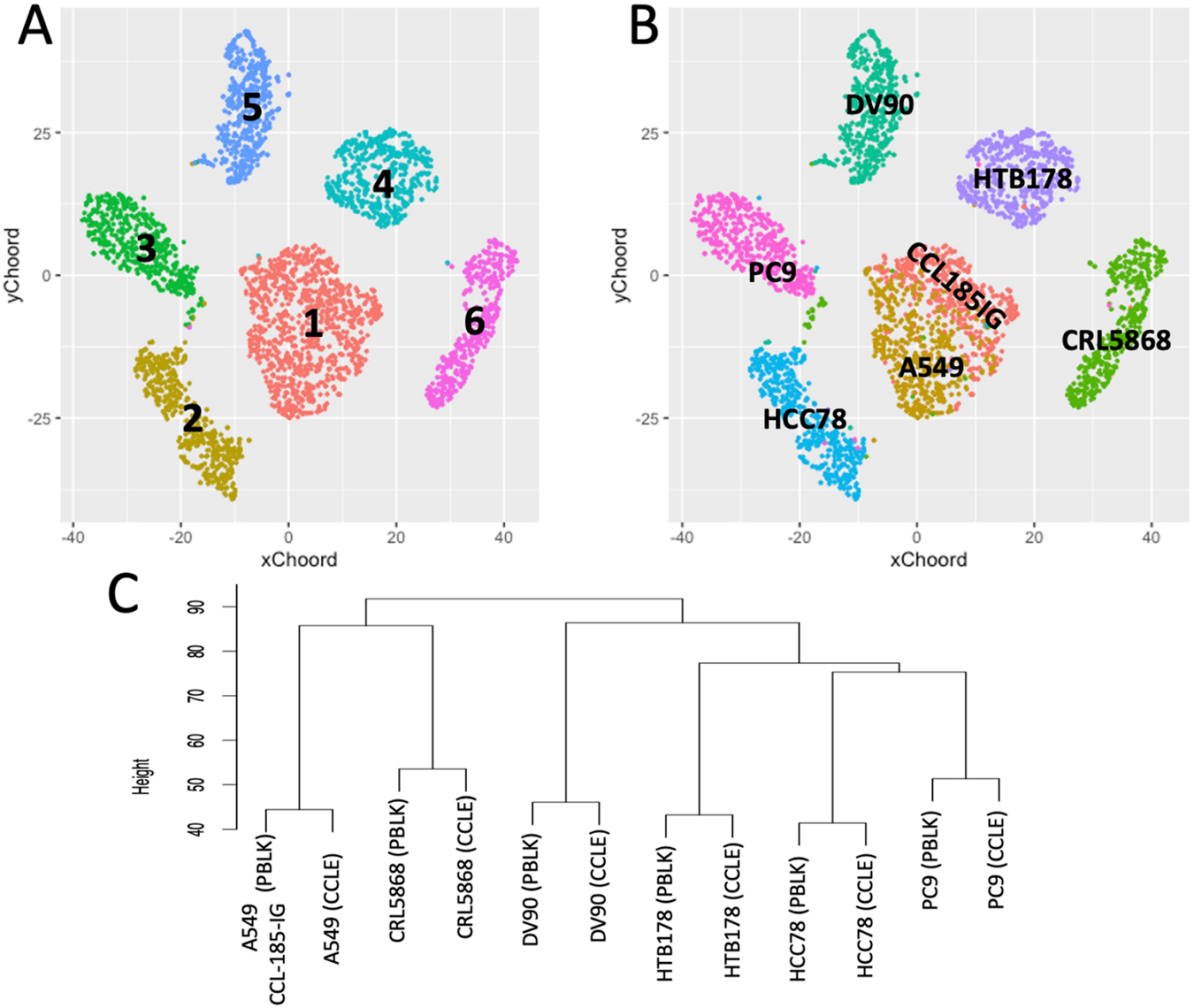
BE1.500 dataset, 500 cells, randomly selected from each of the seven cell lines experiment, were combined in a unique dataset and clustered using Louvain modularity method^33^. (**A**) the clustering results are presented at a resolution of 0.1. (**B**) cell lines association to the clusters. A549 and CCL-185-IG are syngeneic, and despite the expression of ALK in CCL-185-IG, this does not lead to the segregation of A549 and CCL-185-IG into distinct clusters. (**C**) Hierarchical clustering of the clusters pseudo-bulks, generated by aggregating the expression levels of all cells within each cluster, and the bulk transcriptomes of the cell lines downloaded from Cancer Cell Lines Encyclopedia (CCLE). The clustering was done using the best 100 positively expressed markers depicted by COMET software for each cluster.

To ensure that the individual cells in this single cell experiment exhibit traits consistent with the overall features of the original cell lines, we examined the agreement between this dataset and the “bulk” transcriptome of the corresponding cell lines obtained from the Cancer Cell Lines Encyclopedia (CCLE). In particular, we randomly selected 500 cells for each cell line from this experiment, combining them into the BE1-500 dataset^32^. Utilizing the Seurat clustering method^33^, implemented in rCASC^28^, with a resolution of 0.1, the analysis yielded six clusters, as depicted in Fig. 3A. Each cluster is predominantly composed of cells from a specific cell line, except for cluster 1, which incorporates cells from the syngeneic cell lines A549 and CCL-185-IG, Fig. 3B. Using the COMET software^34^, integrated into rCASC, we pinpointed the top 100 gene markers unique to each cluster. Following this, the clusters underwent transformation into pseudo-bulks, using the function bulkClusters implemented in rCASC by consolidating the expression levels of all cells within each cluster for every gene, and the counts were then converted to log_2_CPM. The log_2_TPM expression data for the bulk transcriptome of PC9, HCC78, HTTB178, DV90, CRL5868, and A549 cell lines were sourced from the CCLE database. Genes from both the pseudo-bulks and bulk transcriptome were filtered to encompass only those specific to the clusters identified by the COMET^34^ software. The data underwent hierarchical clustering using the clustering function within the R package (version 4.1.0), employing euclidean distance and average linkage, Fig. 3C. This hierarchical clustering distinctly highlights the alignment of expression profiles between each single-cell pseudo-bulk and the respective cell line transcriptome.

**Fig. 4:**
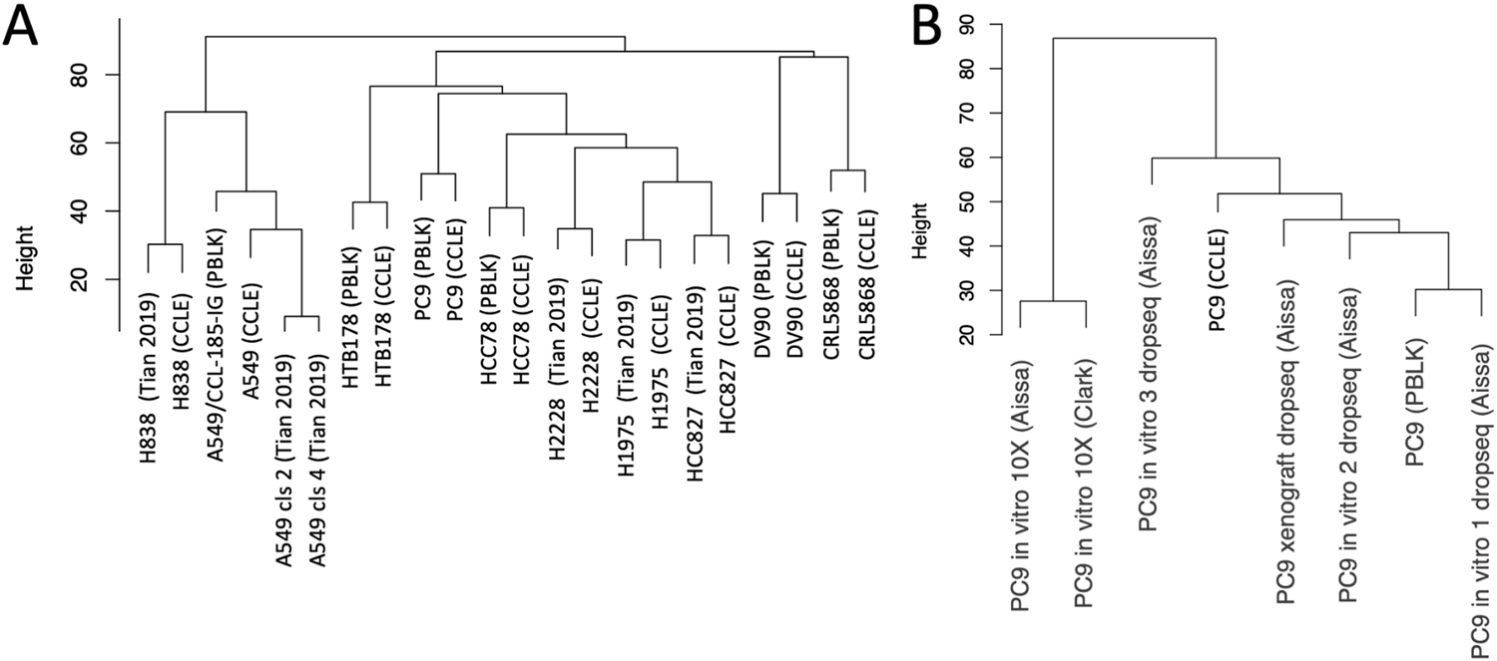
Evaluating the similarity among the BE1-500 pseudo-bulks and the pseudo-bulks generated for lung cancer single cell data^35^ and CCLE cell line bulk transcription profiling. (**A**) Tian’s single cell RNA-seq experiment^35^. This dataset includes: H2228 (PTPN3 KO^33^, EML4-ALK Fusion^40^), H1975 (EGFR L858R and T790M mutations^41^), A549 (KRAS p.G12S, growth and proliferation^14^), H838 (KRASp.G12D^42^) and HCC827 (EGFR L858R mutation^43^) cell lines. (**B**) pseudo-bulk data derived from scRNA-seq published by Aissa *et al*.^36^ and Clark *et al*.^37^.

The current single-cell RNA sequencing experiment can be effectively integrated with prior data^35–37^ obtained from the same lung cancer cell lines, specifically A549 (Fig. 4A) and PC9 (Fig. 4B), as well as with other cell lines characterized by the expression of identical cancer driver genes (Fig. 4A), such as EGFR (H1975, HCC827) and KRAS (H838). Notably, H2228, which harbors the EML4-ALK fusion, clusters together with cell lines expressing mutated EGFR in both pseudo-bulk and CCLE bulk transcriptome analyses, as illustrated in Fig. 4A. This observation aligns with recent findings by Katayama and colleagues^38^, indicating that the adaptive resistance to lorlatinib in ALK-rearranged NSCLC involves EGFR signaling.

## Usage Notes

BE1 10XGenomics count matrices and annotated derivatives dataset^23,25^ includes:

1. The script to run cellranger count (counting.sh), which requires the configuration file multi_gex.csv and the fastq available at GEO repository^25^.
2. This dataset contains for each cell line:
  - QC of the 10XGenomics run (metrics_summary.csv, web_summary.html).
  - The sparse matrices generated by cellranger software.

We have also created an R Shiny App (http://aisc.hpc4ai.unito.it:3838/) that enables the generation of a sparse matrix by blending the seven cell lines at various ratios. The output are sparse matrices in 10XGenomics format, with cell barcodes containing the name of the corresponding cell line (e.g., TCTGCCACATGTGCTA-1_A549). The Shiny App produces several user-defined datasets based on non-overlapping cells characterized by user-defined cell heterogeneity. This feature proves particularly valuable for generating benchmark datasets essential in validating computational tools designed for characterizing cancer heterogeneity through single-cell analysis.

## Data Availability

Cellranger version 7.1.0, which was used to generate count matrices, is available as docker container^39^. All supplementary materials^25,26^ generated using R code, contain a script (command.R) providing all the information needed to reconstruct the supplementary materials.

## References

[1] Tuveson D, Clevers H (2019). Cancer modeling meets human organoid technology. Science.

[2] Navin N (2011). Tumour evolution inferred by single-cell sequencing. Nature.

[3] Patel AP (2014). Single-cell RNA-seq highlights intratumoral heterogeneity in primary glioblastoma. Science.

[4] Li H (2017). Reference component analysis of single-cell transcriptomes elucidates cellular heterogeneity in human colorectal tumors. Nat Genet.

[5] McCray T, Moline D, Baumann B, Vander Griend DJ, Nonn L (2019). Single-cell RNA-Seq analysis identifies a putative epithelial stem cell population in human primary prostate cells in monolayer and organoid culture conditions. Am J Clin Exp Urol.

[6] Heumos L (2023). Best practices for single-cell analysis across modalities. Nat Rev Genet.

[7] De Falco A, Caruso F, Su XD, Iavarone A, Ceccarelli M (2023). A variational algorithm to detect the clonal copy number substructure of tumors from scRNA-seq data. Nat Commun.

[8] Jin Z (2022). Single-cell gene fusion detection by scFusion. Nat Commun.

[9] Zhou Z, Xu B, Minn A, Zhang NR (2020). DENDRO: genetic heterogeneity profiling and subclone detection by single-cell RNA sequencing. Genome Biol.

[10] Muyas, F. *et al*. De novo detection of somatic mutations in high-throughput single-cell profiling data sets. *Nat Biotechnol*, 10.1038/s41587-023-01863-z (2023). 10.1038/s41587-023-01863-z PMC11098751 37414936

[11] Zeng, P., Ma, Y. & Lin, Z. scAWMV: an adaptively weighted multi-view learning framework for the integrative analysis of parallel scRNA-seq and scATAC-seq data. *Bioinformatics* **39**, 10.1093/bioinformatics/btac739 (2023). 10.1093/bioinformatics/btac739 PMC9805575 36383176

[12] Weber LM (2019). Essential guidelines for computational method benchmarking. Genome Biol.

[13] Simonetti S (2010). Detection of EGFR mutations with mutation-specific antibodies in stage IV non-small-cell lung cancer. J Transl Med.

[14] Yoon YK (2010). KRAS mutant lung cancer cells are differentially responsive to MEK inhibitor due to AKT or STAT3 activation: implication for combinatorial approach. Mol Carcinog.

[15] Negrao MV (2020). Molecular Landscape of BRAF-Mutant NSCLC Reveals an Association Between Clonality and Driver Mutations and Identifies Targetable Non-V600 Driver Mutations. J Thorac Oncol.

[16] Bose R (2013). Activating HER2 mutations in HER2 gene amplification negative breast cancer. Cancer Discov.

[17] Cerqua, M. *et al*. MET∆14 promotes a ligand-dependent, AKT-driven invasive growth. *Life Sci Alliance* **5**, 10.26508/lsa.202201409 (2022). 10.26508/lsa.202201409 PMC9152130 35636967

[18] Nosi, V. *et al*. MET Exon 14 Skipping: A Case Study for the Detection of Genetic Variants in Cancer Driver Genes by Deep Learning. *Int J Mol Sci* **22**, 10.3390/ijms22084217 (2021). 10.3390/ijms22084217 PMC8072630 33921709

[19] Davies KD (2012). Identifying and targeting ROS1 gene fusions in non-small cell lung cancer. Clin Cancer Res.

[20] Enuameh, M. S. *et al*. Developing isogenic cell models with CRISPR: an EML4-ALK fusion NSCLC cell line. *Nature* (2019).

[21] Chen J (2022). Single-cell DNA-seq depicts clonal evolution of multiple driver alterations in osimertinib-resistant patients. Ann Oncol.

[22] Dong X (2023). NetBID2 provides comprehensive hidden driver analysis. Nat Commun.

[23] Arigoni MR (2023). GEO.

[24] Arigoni MR (2023). NCBI SRA.

[25] Calogero R, Riccardo F, Arigoni M, Ratto ML, Alessandri L (2023). Figshare.

[26] Calogero R, Riccardo F, Arigoni M, Ratto ML, Alessandri L (2023). Figshare.

[27] Han H (2018). TRRUST v2: an expanded reference database of human and mouse transcriptional regulatory interactions. Nucleic Acids Res.

[28] Mandreoli P, Alessandri L, Calogero RA, Tangaro MA, Zambelli F (2023). Using “Galaxy-rCASC”: A Public Galaxy Instance for Single-Cell RNA-Seq Data Analysis. Methods Mol Biol.

[29] Contaldo SG, Alessandri L, Colonnelli I, Beccuti M, Aldinucci M (2023). Bringing Cell Subpopulation Discovery on a Cloud-HPC Using rCASC and StreamFlow. Methods Mol Biol.

[30] Alessandri, L. *et al*. rCASC: reproducible classification analysis of single-cell sequencing data. *Gigascience* **8**, 10.1093/gigascience/giz105 (2019). 10.1093/gigascience/giz105 PMC6732171 31494672

[31] Ordonez-Rueda D (2020). Apoptotic Cell Exclusion and Bias-Free Single-Cell Selection Are Important Quality Control Requirements for Successful Single-Cell Sequencing Applications. Cytometry A.

[32] Calogero R, Calogero L (2023). Figshare.

[33] Satija R, Farrell JA, Gennert D, Schier AF, Regev A (2015). Spatial reconstruction of single-cell gene expression data. Nat Biotechnol.

[34] Delaney C (2019). Combinatorial prediction of marker panels from single-cell transcriptomic data. Mol Syst Biol.

[35] Tian L (2019). Benchmarking single cell RNA-sequencing analysis pipelines using mixture control experiments. Nat Methods.

[36] Aissa AF (2021). Single-cell transcriptional changes associated with drug tolerance and response to combination therapies in cancer. Nat Commun.

[37] Clark IC (2023). Microfluidics-free single-cell genomics with templated emulsification. Nat Biotechnol.

[38] Katayama Y (2023). Adaptive resistance to lorlatinib via EGFR signaling in ALK-rearranged lung cancer. NPJ Precis Oncol.

[39] Calogero, R. Cellranger 7.1.0 docker image. *Docker.com* (2023).

[40] Isozaki H (2016). Non-Small Cell Lung Cancer Cells Acquire Resistance to the ALK Inhibitor Alectinib by Activating Alternative Receptor Tyrosine Kinases. Cancer Res.

[41] Zhao BX (2015). Establishment and biological characteristics of acquired gefitinib resistance in cell line NCI-H1975/gefinitib-resistant with epidermal growth factor receptor T790M mutation. Mol Med Rep.

[42] Wang Z, Yin M, Chu P, Lou M (2019). STAT3 inhibitor sensitized KRAS-mutant lung cancers to RAF inhibitor by activating MEK/ERK signaling pathway. Aging (Albany NY).

[43] Tang ZH (2018). Increased Expression of IRE1alpha Associates with the Resistant Mechanism of Osimertinib (AZD9291)-resistant non-small Cell Lung Cancer HCC827/OSIR Cells. Anticancer Agents Med Chem.

